# The effect of patient age at intervention on risk of implant revision after total replacement of the hip or knee: a population-based cohort study

**DOI:** 10.1016/S0140-6736(17)30059-4

**Published:** 2017-04-08

**Authors:** Lee E Bayliss, David Culliford, A Paul Monk, Sion Glyn-Jones, Daniel Prieto-Alhambra, Andrew Judge, Cyrus Cooper, Andrew J Carr, Nigel K Arden, David J Beard, Andrew J Price

**Affiliations:** a Nuffield Department of Orthopaedics, Rheumatology, and Musculoskeletal Science, NIHR Biomedical Research Unit, University of Oxford, Oxford, UK; b Arthritis Research UK Centre for Sport, Exercise and Osteoarthritis, University of Oxford, Oxford, UK; c GREMPAL Research Group, Idiap Jordi Gol and CIBERFes, Universitat Autonoma de Barcelona and Instituto de Salud Carlos III, Barcelona, Spain; d MRC Lifecourse Epidemiology Unit, University of Southampton, Southampton, UK; e NIHR CLAHRC Wessex Methodological Hub, University of Southampton, Southampton, UK

## Abstract

**Background:**

Total joint replacements for end-stage osteoarthritis of the hip and knee are cost-effective and demonstrate significant clinical improvement. However, robust population based lifetime-risk data for implant revision are not available to aid patient decision making, which is a particular problem in young patient groups deciding on best-timing for surgery.

**Methods:**

We did implant survival analysis on all patients within the Clinical Practice Research Datalink who had undergone total hip replacement or total knee replacement. These data were adjusted for all-cause mortality with data from the Office for National Statistics and used to generate lifetime risks of revision surgery based on increasing age at the time of primary surgery.

**Findings:**

We identified 63 158 patients who had undergone total hip replacement and 54 276 who had total knee replacement between Jan 1, 1991, and Aug 10, 2011, and followed up these patients to a maximum of 20 years. For total hip replacement, 10-year implant survival rate was 95·6% (95% CI 95·3–95·9) and 20-year rate was 85·0% (83·2–86·6). For total knee replacement, 10-year implant survival rate was 96·1% (95·8–96·4), and 20-year implant survival rate was 89·7% (87·5–91·5). The lifetime risk of requiring revision surgery in patients who had total hip replacement or total knee replacement over the age of 70 years was about 5% with no difference between sexes. For those who had surgery younger than 70 years, however, the lifetime risk of revision increased for younger patients, up to 35% (95% CI 30·9–39·1) for men in their early 50s, with large differences seen between male and female patients (15% lower for women in same age group). The median time to revision for patients who had surgery younger than age 60 was 4·4 years.

**Interpretation:**

Our study used novel methodology to investigate and offer new insight into the importance of young age and risk of revision after total hip or knee replacement. Our evidence challenges the increasing trend for more total hip replacements and total knee replacements to be done in the younger patient group, and these data should be offered to patients as part of the shared decision making process.

**Funding:**

Oxford Musculoskeletal Biomedical Research Unit, National Institute for Health Research.

## Introduction

Hip and knee replacements have been routinely done for the treatment of end-stage arthritis over the past 40 years;^1^, ^2^ 76 000 total hip replacements and 82 000 total knee replacements were done in 2014 in the UK alone,^3^ with the greatest increase in the number of total knee replacements in recent years. The outcomes of joint replacements are determined in several different ways, including mortality^4^, ^5^ and morbidity rates after surgery, functional outcome and satisfaction recorded as patient-reported outcome scores,^6^ and by rates of failure of the implant leading to revision surgery.^3^, ^7^ Total hip replacement and total knee replacement have demonstrated improved function,^8^ reduced pain, and improved quality of life^9^ for patients, and are cost-effective.^9^ Predictions are that in the next 10–20 years primary joint replacement rates will substantially increase, not only as a consequence of an ageing population, but also because of increasing use in patients younger than age 60 years,^10^ who currently represent 15% of the entire population undergoing surgery, but might increase in the future.^11^

This rise in the number of patients younger than 60 years undergoing surgery is a concern because joint registries reveal that 10-year revision rates in this group are higher than for older age groups.^3^ For all patients, the decision to have surgery is largely based on the balance between potential risk and benefit. The James Lind Alliance Priority Setting Partnership, a public–patient involvement group, has established that the relation between timing of joint replacement and best outcome is one of the most significant concerns for patients with osteoarthritis. This is of particular importance in determining optimum timing for surgery in younger patients, where they can be expected to potentially outlive their primary replacement. Therefore, the length of time a joint replacement will last (before requiring revision) becomes a major factor in deciding whether to proceed with surgery. The most widely used and quoted data for risk of revision come from joint registry reports, but are often limited to 10 years of follow-up.^3^, ^7^, ^12^ Other studies with longer patient follow-up (greater than 20 years) are frequently restricted to specific prostheses or small populations, without specific focus on the results from patients younger than 60 years at implantation.^13^, ^14^ Therefore, for these patients, information about implant revision rates tends to be restricted to 10 years, and although worse than those seen for patients older than 60,^3^, ^9^, ^15^ might not truly reflect the risk of revision over the longer timeframe. The decision making process for younger patients is hence not fully informed, and could lead to inappropriate election to undergo joint replacement.^15^, ^16^

**Research in context**

**Evidence before this study**

We searched Medline, Embase, and the Cochrane Library up to Nov 2015 for all studies investigating lifetime risk of revision in total hip and total knee replacement or arthroplasty. Whilst publications were identified for lifetime risk of undergoing primary joint replacement of the hip or knee, no such studies were found for lifetime risk of revision surgery. Review of National Joint Registries reports (England & Wales, Australia, New Zealand, Sweden, Norway, Denmark) revealed publication of annual survival incidences but not lifetime risk predictions.

**Added value of this study**

This study proposes the use of lifetime revision risk predictions, based on a large dataset, as a useful and novel tool for both clinicians and patients. Although data are available that describe the lifetime risk of need for primary joint replacement, data have not been published for revision risk following primary procedure. Before this study, revision rates have been quoted as survival incidences (usually at 10 years) however the context of a 10-year revision risk is very different for young patients compared to those of greater age, for whom 10 years could represent the majority of their life-expectancy. This study allows patients to understand the risk in the context of their predicted life-expectancy, and as such, better inform their decision to undergo joint replacement surgery.

**Implications of all the available evidence**

Previous study data, increasingly based upon national registers, showed that young patients are at a higher risk of revision than are older patients; this study expands upon this problem to better highlight how much greater this risk is over a patient's lifetime and to put this into a statistic that is easier to explain to patients considering surgery. The number of younger patients undergoing joint replacement surgery is increasing and it is crucial that their decision to undergo surgery is based on best available, personalised evidence.

There is a clear need for more representative long-term data that could be used to inform patients of the risk of revision surgery. One approach that has not previously been used comes from combining data from the Clinical Practice Research Datalink, a database that contains long-term data for joint replacement that spans over 20 years, and adopting different methods of analysis that are new to this discipline. The notion of lifetime risk describes the probability (expressed as a percentage) of an event or disease occurring over the course of a lifetime. It has been used in oncology research, and infrequently in musculoskeletal literature,^17^, ^18^, ^19^ but has never been used to assess the lifetime risk of revision surgery after joint replacement. Lifetime risk data is useful to patients, clinicians, and health-care planners alike as it provides a simple idea to convey to patients and is easier to understand than time-dependent incidence rates (such as 10-year risk of revision),^20^ which are commonplace both in the explanation of revision risks to patients undergoing primary joint replacement and in the assessment of prosthesis longevity.^3^

The aim of this study was to determine age adjusted estimates of lifetime risk of undergoing a revision procedure after primary total hip replacement or total knee replacement with data from the Clinical Practice Research Datalink and Office for National Statistics.

## Methods

### Data Sources

Participant data were obtained from the Clinical Practice Research Datalink (CPRD), formerly known as the General Practice Research Database. The CPRD consists of the computerised primary care medical records of all patients attending a selection of general practitioners in the UK. This population of 6·5 million patients is taken from 433 contributing practices chosen to be representative of the wider UK population;^21^ therefore the CPRD consists of entire general practice populations rather than probability-based samples of patients.

Each patient is registered at one practice, which stores both primary care and hospital episode information. The universal health-care system in the UK is dependent on primary care for referrals and funding of hospital episodes, and therefore the CPRD is a detailed record of both primary and secondary care. The CPRD dataset for each patient contains all clinical and referral events in both primary and secondary care, comprehensive demographic information, prescription data, and hospital admissions data. Data are stored with Read and Oxford Medical Information Systems (OXMIS) codes for diseases that are cross-referenced to the International Classification of Diseases (ICD-9). Read codes are used as the standard clinical terminology system within UK primary care. Only practices that pass quality control are used as part of the CPRD database. Deleting or encoding personal and clinic identifiers ensures confidentiality of information. The CPRD is administered by the Medicines and Healthcare Products Regulatory Agency (MHRA).

### Population

All patients in the database with a diagnostic code for primary total hip or knee replacement from Jan 1, 1991, until Aug 10, 2011, were identified. Read/OXMIS codes were used to identify primary replacements and subsequent revision surgeries (appendix). Patients were included in the analysis if aged 50 years or over at the time of index primary joint replacement procedure. Participant demographics including age and sex were collated. Sex-specific all-cause mortality data was obtained from the Office for National Statistics (ONS)^22^ for Jan 2, 1991, to Dec 31, 2011.

### Analysis

Data from the CPRD were aggregated into single year intervals by age at the time of index procedure (primary joint replacement) and then subdivided into hip or knee replacement, and by sex. Age was defined as age at last birthday, starting at 50 years; consistent definitions were applied to death data and timing of any surgery. Person–time incidence rates for revision surgery were calculated by dividing the count of revision replacements by the cumulative time with primary implant.

All-cause mortality rates taken from the ONS data were applied to this population to generate the number of implant-years for each interval—ie, the period of exposure to potential revision surgery (eg, 100 patients with 1·0% mortality would generate 99 implant-years for the first year interval). All-cause mortality and annual incidence rates were applied as multiple decrements at 1-year intervals. The total number of counts for predicted revisions was summed and divided by the population to produce an estimate of lifetime risk for patients undergoing surgery between the ages of 50 and 100 years (in 5-year age bands for ages 50–54 years through to 85 years and older).

Revision incidence rates were also applied to the censored (ie, implant in situ at the end of the study period) and lost-to-follow-up populations to generate an adjusted revision–incidence (lost and censored population) which was also then adjusted for ONS mortality rates in the same way. Lifetime risk of revision surgery was calculated by grouping the 1-year intervals into 5-year age-bands.^23^ An actuarial life-table method was applied, as previously described, to a hypothetical population of the same magnitude as the subgroup under investigation. Count data for incidence of revision surgery was assumed to be a count-random variable and as such a Poisson distribution was used to calculate 95% CI.^17^Smoothed hazard plots showing instantaneous risk of revision (risk of revision following a given period of implant survival) were generated for both sexes.

All statistical analyses were done with Stata (Statacorp. 2014; Stata Statistical Software: release version IC 13.1. College Station, Texas, USA) and Microsoft Excel 2011 (Microsoft Corporation, Redmond, WA).

The CPRD Group has obtained ethical approval from a National Research Ethics Service Committee (NRES) for all purely observational research with anonymised CPRD data—namely, studies that do not include patient involvement. The study has been approved by ISAC (Independent Scientific Advisory Committee) for MHRA Database Research, protocol number 11_050A.

### Role of the funding source

Support was received from the National Institute for Health Research (NIHR) Oxford Musculoskeletal Biomedical Research Unit, the sponsor had no role in study design, data collection, data analysis, data interpretation, or report preparation. The corresponding author (AJP) had full access to all the data in the study and AJP had final responsibility for the decision to submit for publication.

## Results

**Figure 1:**
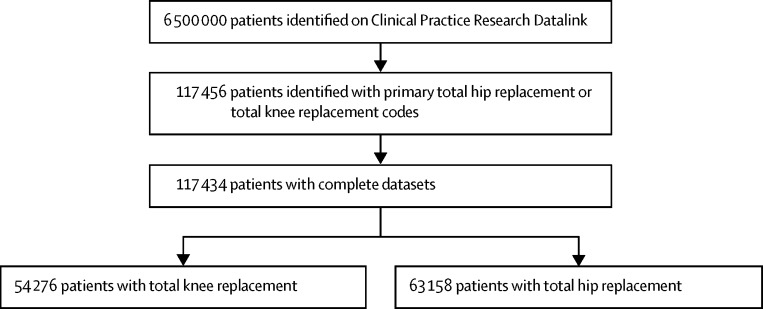
trial profile

Between Jan 1, 1991, and Dec 31, 2011, 117 434 patients were identified from the database as having undergone a total hip replacement (n=63 158) or total knee replacement (n=54 276) during the study period (figure 1). The mean age of patients undergoing joint replacement was 69·4 years (SD 11·1) for hip and 70·1 years (9·6) for knee replacement; 15% of patients were aged 50–60 years at time of surgery in both the hip and knee replacement groups, and 15% were older than 79 years in both groups. The number of women undergoing surgery was greater for both hip and knee replacement (table 1). Mean total hip replacement follow-up was 5·8 years (range 0·0–23·1, median 4·9), and for total knee replacement was 5·2 years (0·0–22·5, 4·5).

**Table 1:** Demographic data

	**Female**	**Male**	**Total**
**Total hip replacement**			
N (%)	39 289 (62%)	23 869 (38%)	63 158
Mean age in years (SD)	70·4 (11·1)	67·7 (11·0)	69·4 (11·1)
**Total knee replacement**			
N (%)	31 682 (59·5%)	22 594 (41·5%)	54 276
Mean age in years (SD)	70·7 (9·6)	69·4 (9·4)	70·1 (9·6)

**Figure 2:**
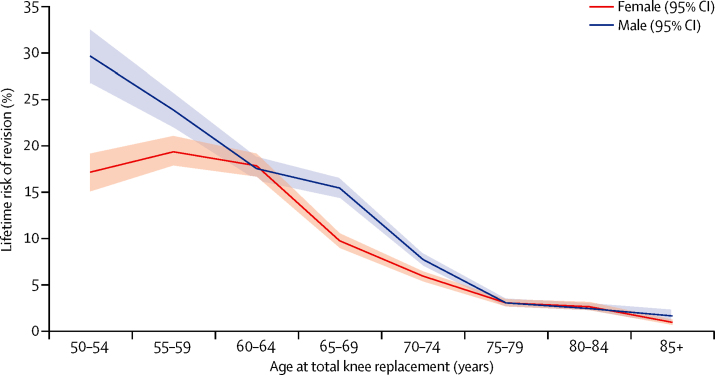
Lifetime risk of revision after total hip replacement Plot showing estimates of lifetime risk of total hip replacement revision against age at the time of total hip replacement primary surgery (in 5-year age bands) and stratified by sex (results adjusted for lost and censored population).

**Figure 3:**
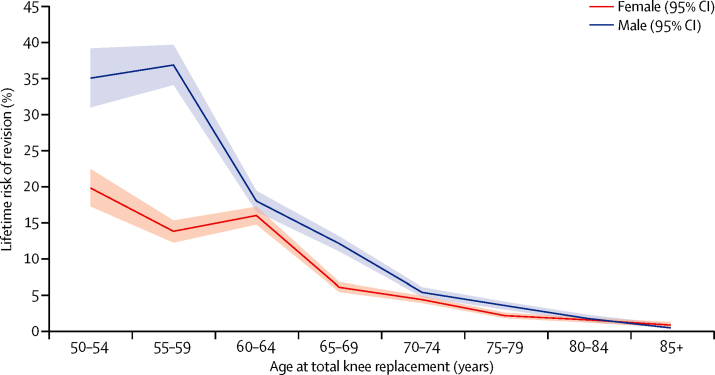
Lifetime risk of revision after total knee replacement Plot showing estimates of lifetime risk of total knee replacement revision against age at the time of primary total knee replacement surgery (in 5-year age bands) and stratified by sex (results adjusted for lost and censored population).

10-year implant survival rate was 95·6% (95% CI 95·3–95·9) and 20-year implant survival rate was 85% (83·2–86·6) for total hip replacement (table 2). 10-year implant survival rate was 96·1% (95·8–96·4) and 20-year rate was 89·7% (87·5–91·5) for total knee replacement (table 3). In both types of replacement, implant survival over time was higher for female patients and older patients (Figure 2, Figure 3, appendix), with the lowest implant survival rates seen in patients in their 50s at the time of index surgery.

**Table 2:** Patient survival at 5, 10, 15, and 20 years after total hip replacement

	**Total**	**Implants revised**	**Deaths**	**Lost to follow-up or censored**	**Cumulative implant survival rate (95% CI)**	**Lost and censored population adjusted survival**
5	37 066	144	991	4845	0·979 (0·9779–0·9804)	0·977
10	13 203	62	514	2330	0·956 (0·9534–0·9585)	0·950
15	3312	33	158	691	0·910 (0·9029–0·9157)	0·893
20	444	3	19	199	0·850 (0·8323–0·8663)	0·812

**Table 3:** Patient survival at 5, 10, 15, and 20 years after total knee replacement

	**Total**	**Implants revised**	**Deaths**	**Lost to follow-up or censored**	**Cumulative implant survival rate (95% CI)**	**Lost and censored population adjusted survival**
5	30 056	89	1427	5648	0·9798 (0·9784–0·9812)	0·977
10	8261	25	495	2128	0·9612 (0·9583–0·9639)	0·953
15	1717	12	155	523	0·9294 (0·9217–0·9364)	0·912
20	152	0	12	72	0·8969 (0·8745–0·9154)	0·862

The estimated lifetime risk of revision (LTRR) increased with decreasing age at the time of primary surgery for both hip and knee replacements (Figure 2, Figure 3). For patients aged 70 years at implantation (mean age of implantation) the LTRR was between 4·4% and 7·7% (highest in male patients with total hip replacement**).** Older than this age, LTRR reduced with time for both hip and knee replacement, and was consistent between sexes. For patients aged between 60 and 70 years at the time of primary surgery, LTRR increased with decreasing age, reaching approximately 15% for both hip and knee replacement at 60 years, with greater risk in male than in female patients. For women between the ages of 50 and 60 years at primary surgery, the LTRR does not change a great deal for total hip replacement and increases by a few percentage points for total knee replacement. However, a significant increase in LTRR was seen in younger men, with values of 29·6% (95% CI 26·6–32·6) for hip and 35·0% (30·9–39·1) for knee replacement seen for the youngest patient group (50–54 years).

14% (n=22 122) of the study population died during the study period, with a mean age of 75·3 years (SD 7·9) at time of surgery and a mean age of 80·8 years (SD 8·11) at death. Of these patients, 98% (n=21 624) died with their primary implant still in situ.

**Figure 4:**
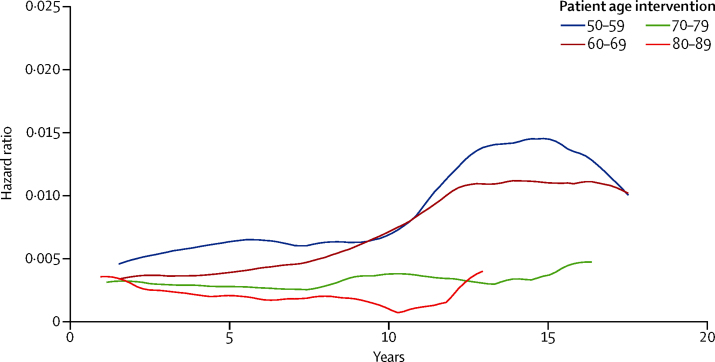
Smoothed hazard curve of revision risk in female patients by age Instantaneous risk of revision for a given length of implant survival, stratified by age at time of primary total hip replacement or total knee replacement (in 10-year age-bands).

**Figure 5:**
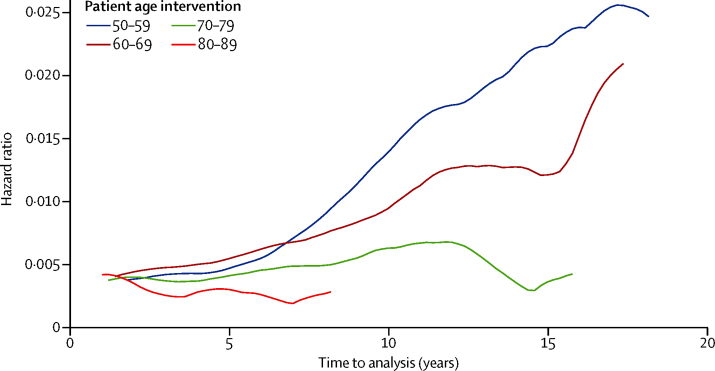
Smoothed hazard curve of revision risk in male patients by age Instantaneous risk of revision for a given length of implant survival, stratified by age at time of primary total hip replacement or total knee replacement (in 10-year age-bands).

The timing of revision surgery shows a peak incidence within 5 years of primary implantation in all age ranges, with a mean time to revision surgery of 6·56 years (95% CI 6·05–7·08) in hip and 4·55 (4·07–5·02) in knee replacement for patients aged 50–59 years at initial surgery and 4·08 (3·73–4·39) for hip and 3·57 (3·26–3·88) for knee replacement in their eighth decade. The smoothed hazard plots in Figure 4, Figure 5 show consistently higher revision risks for men and younger patients at all timepoints. These graphs also show that the trends of timing to revision surgery are similar across all age bands, with the exception of the most elderly patient groups, in whom follow-up is limited by life expectancy.

The mean annual rate of patients lost to follow-up (excluding censored patients) is 2·2% for total hip replacement (95% CI 2·0–2·4) and 1·8% for total knee replacement (1·7–2·0). Adjustment for these lost and censored patients increased the estimate of LTRR similarly in each age group.

The lost and censored subpopulations were analysed at a number of timepoints and found to be consistently of the same demographics as the remaining population for that period.

## Discussion

Our results show that for patients who are younger than 60 years at primary surgery, their lifetime risk of revision increases significantly, reaching up to one in three in those patients aged 50–55 years. These figures are in contrast to older patients for whom our data show that for patients undergoing hip or knee replacement at or over 70 years of age, the lifetime risk of requiring revision surgery is between 1% and 6%; this estimate applies to about half of patients undergoing this type of surgery. Time to revision surgery reaches a peak around 5 years after implantation, with over half of revisions occurring within 6 years after primary surgery for all age groups. As far as we are aware this is the first time this methodology has been applied to joint replacement revision surgery and it emphasises the dramatic effect age has on risk of revision after surgery.

Our data are supported by previously published data in this area where an increased revision rate in younger patients has been identified.^3^, ^24^, ^25^ However, most population studies that specifically address this issue are based on 10-year follow-up data from registries. In fact, no previous studies have quantified the risk of revision over the patient's life and examined when revision is likely to be done. Our study not only highlights a lifetime revision risk for patients under 60 years at intervention of up to 35%, with risk of revision higher than in other age-groups at all timepoints, but also that the time to revision in many cases is within 5 years, which itself carries an increased re-revision risk. ^3^ As a result, young patients are likely to spend many more years than previously expected with a revision implant, which carries with it poor outcomes.^26^

We noted that sex has a significant influence on the estimated lifetime revision rate for both total hip replacement and total knee replacement. Below age 70 years, men have a consistently higher estimated LTRR. The effects are seen most dramatically in the youngest age group (50–55 years) in whom LTRR for men is 24% after total knee replacement, about 1·7 times greater than for women undergoing the same procedure, with similar trend after total hip replacement. These same trends are also demonstrated by the smoothed hazard plots (Figure 4, Figure 5) with the instantaneous risk of revision in these groups being raised at all timepoints.

Although no information exists for lifetime risk data for revision joint replacement, the technique has been previously used to study primary joint replacement for hip and knee osteoarthritis; lifetime risk for the development of osteoarthritis is estimated at 25% for the hip and 45% for the knee^18^ and lifetime risk of undergoing total hip replacement is 11·6% for women and 7·1% for men, and for total knee replacement 10·8% for women and 8·1% for men.^17^

As well as presenting the novel use of lifetime revision estimates, we also did implant survival analysis on the CPRD dataset. This analysis allowed us to compare and validate our data against published work from the joint registries that use the same methodology. The UK National Joint Registry (UK NJR) published figures for 10-year revision risk were 5·75% for total hip and 4·47% for total knee replacement, which showed similar results as the implant survival analysis results in this study (5·0% for hip and 4·7% for knee replacement),^3^ with similar trends for reducing implant survival in patients aged under 60 years over a 10-year period. However, we believe our data suggest that 10-year implant survival does underestimate the scale of the problem for the younger patient. For instance, in a patient with a 10-year life expectancy, an estimate of the potential 10-year survival of the implant provides good insight into the likely chance of undergoing revision. However, for a patient under 60 years, who might live for another 30–40 years, 10-year implant survival could underestimate exposure to the real risk of revision. A much more representative figure is the estimate of lifetime risk (in this case based on a dataset with up to 20 years' follow-up).

The study has some limitations. It focuses on implant survival as an indicator of successful outcome after joint replacement; we acknowledge that a patient's outcome after surgery is more complex than this simple measure. Patient reported outcomes, morbidity data, and mortality data are equally as important to patients. Ideally, data for these factors and LTRR would all be available to patients who are deciding whether or not to undergo surgery. Lifetime risk estimates in this large population study might be affected by the smaller numbers in the stratified age and sex subgroups in the final follow-up periods. As such, where appropriate, our estimates were based on 15-year follow-up data to maintain population subgroup size.

This study does not include data relating to the indication for surgery or implant type (including metal-on-metal or ceramic bearing surfaces), whereas evidence exists that these factors can contribute to variations in implant survival.^3^, ^7^ To limit these effects, patients under the age of 50 years were excluded to avoid including the more complex pathology seen in younger patients who require surgery. An analysis of annual revision rates across the 20-year follow-up period was consistent, suggesting the effect of changing trends in implant use was minimal. Laterality data for each patient was not available, nor was coding for previous contralateral procedure. Although this omission should not have a large effect on the lifetime risk estimations, it was not possible to adjust for bilateral disease as a potential risk factor for revision. The first chronological codes for primary surgery and revision were consistently taken for each patient, and this might have underestimated how quickly these patients underwent revision.

The lifetime risk calculation is a standard method permitting multiple decrements to account for competing risks (all-cause mortality), but it does not afford the flexibility of model-based methods in dealing with predictor variables. In this study, the lifetime risk calculation is based on follow-up data of up to 20 years, and could underestimate the revision rates seen, especially in younger patients in whom predicted life-expectancy exceeds 20 years. The use of all-cause mortality data from the ONS does not account for the reduced mortality rates seen in patients presenting for joint replacement compared with the general population,^27^ and might overestimate mortality rates and subsequently over-estimate LTRR estimates.

The study also showed that some patients were lost to follow-up; these patients were subsequently accounted for and reintroduced into the population after having the same revision incidence applied to them found at the time they left the study, and the same principle was applied to censored patients. Previous studies have advocated treating patients in these loss-to-follow-up groups with higher failure rates than those seen in the surveyed population; this is often the consequence of analysis of single-centre series. The nature of the CRPD is such that patients are lost to follow-up if they move geographical location and subsequently out of the catchment of their primary care practice; census data suggest people are more likely to move when medically well. Given the nature of the CPRD population, care should be taken when extrapolating these results to other populations in which health-care behaviours and practices differ from those in the UK.

However, the strength of this study is its population-level data and subsequently large sample. In addition, the CPRD represents a large population dataset selected to be representative of the UK as a whole;^21^ as a consequence, results derived from this dataset will be less at risk of confounding factors often found in smaller datasets and those collected from smaller regions where local factors (including demographic, socioeconomic, and referral thresholds) could vary.

This work sheds new light on the risk of revision surgery for patients younger than age 60 years. Although it has been previously established that this group of patients have a higher 10-year revision rate than patients older than 60 years, we believe that the true risk to patients is much higher than previously thought. For patients under 60 years of age, the lifetime risk of revision increases to a third, with the highest levels of revision seen in men between the ages of 50–55 years. These higher lifetime risks are paired with higher risks of revision at all timepoints and short mean times to revision, meaning a patient in their 50s with a potential life expectancy of more than 30 years could spend many years living with a revision joint replacement with limited functional ability. On a broader level with the numbers of joint replacements increasing year on year this issue will create a significant health economic burden for any health-care system.^28^ In patients older than 60 years at first intervention, the risk of revision decreases and by age 70 years the likelihood of revision surgery in some patients is less than 1 in 20. In this age group, 95% of patients will retain their prosthesis, suggesting that long term revision rates are not as high as they seem to be.^3^, ^29^

At a personal level these new data have significant implications for patients younger than age 60, who should consider the possibility of living with a revision procedure for many years if they undergo total hip replacement or total knee replacement and subsequently require early revision. Patients who are considering undergoing joint replacement should balance the potential benefits of an improvement in their quality of life against the potential risks of the intervention: death, medical complications, infection, poor functional outcome, and the need for revision surgery. Patients have indicated that they require improved information about these outcomes, particularly in relation to deciding on the correct time to have surgery. A patient's age and sex affect these outcomes and therefore might influence their decision. Patients are most often informed about risk of revision in terms of the likely 10-year survival of their implant, which can be an abstract and potentially confusing idea.^20^ To be able to answer these concerns in as accurate and clear a form as possible is important to provide useful information to aid patient decision making. We believe that an estimate of the lifetime risk of revision is likely to be a valuable addition to the decision making process, and is particularly relevant given the findings of this study, in which differences in outcome highlight the requirement for a more personalised approach to estimating potential risks and benefits for patients who are considering this procedure.

**This online publication has been corrected. The corrected version first appeared at thelancet.com on April 6, 2017**
