# Department of Error

**DOI:** 10.1016/S0140-6736(17)30936-4

**Published:** 2017-04-08

*Bayliss LE, Culliford D, Monk AP, et al. The effect of patient age at intervention on risk of implant revision after total replacement of the hip or knee: a population-based cohort study.* Lancet *2017; **389:** 1424–30*—In the Summary of this Article, the funding section was missing. This Article should have been published with an Open Access copyright licence. This correction was made to the online version as of April 6, 2017, and the printed Article is correct.

