# Supplementary material for: The effect of patient age at intervention on risk of implant revision after total replacement of the hip or knee: a population-based cohort study
Source: Lancet. 2017 Apr 8;389(10077):1424–30. doi: 10.1016/S0140-6736(17)30059-4 (PMC5522532; doi:10.1016/S0140-6736(17)30059-4)

THE LANCET

Supplementary appendix

This appendix formed part of the original submission and has been peer reviewed. We post it as supplied by the authors.

Supplement to: Bayliss LE, Culliford D, Monk AP, et al. The effect of patient age at intervention on risk of implant revision after total replacement of the hip or knee: a population-based cohort study. *Lancet* 2017; published online Feb 13. http://dx.doi.org/10.1016/S0140-6736(17)30059-4.

CPRD Medical Codes

Medical diagnosis codes for hip osteoarthritis

medcode	readcode	readterm
1104	N053512	Hip osteoarthitis NOS
2209	N05z511	Hip osteoarthritis NOS
6812	N05zJ00	Osteoarthritis NOS, of hip

Medical diagnosis codes for knee osteoarthritis

medcode	readcode	readterm
665	N05z611	Knee osteoarthritis NOS
2487	N05zL00	Osteoarthritis NOS, of knee

Medical diagnosis codes for hip replacement

medcode	readcode	readterm
394	7K22z00	Total prosthetic replacement of hip joint NOS
2224	7K20.1G	THR - Total prosthetic replacement of hip joint using cement
5481	7K20.00	Total prosthetic replacement of hip joint using cement
2734	7K22.12	THR - Other total prosthetic replacement of hip joint
9762	7K22.00	Other total prosthetic replacement of hip joint
33439	7K22000	Primary total prosthetic replacement of hip joint NEC
589	7K21.17	THR - Total prosthetic replacement hip joint without cement
18442	7K21.00	Total prosthetic replacement of hip joint not using cement
16671	7K20.13	Charnley total replacement of hip joint using cement
10856	7K20000	Primary cemented total hip replacement
28468	7K20.14	Exeter total replacement of hip joint using cement
17860	7K20.11	Arthroplasty of hip joint using cement

47483	7K21000	Primary uncemented total hip replacement
38001	7K20y00	Total prosthetic replacement of hip joint using cement OS
10348	7K20300	Primary hybrid total replacement of hip joint NEC
47812	7K20z00	Total prosthetic replacement of hip joint using cement NOS
37631	7K22y00	Other specified total prosthetic replacement of hip joint
29977	7K20.1E	Stanmore total replacement of hip joint using cement
38332	7K20.17	Furlong total replacement of hip joint using cement
38347	7K21z00	Total prosthetic replacement hip joint not using cement NOS
52714	7K20011	Charnley cemented total hip replacement
6013	7K20.1C	Muller total replacement of hip joint using cement
36590	7K20.18	Howse total replacement of hip joint using cement
47735	7K21.12	Furlong total replacement of hip joint not using cement
71351	7K20.12	Aufranc total replacement of hip joint using cement
47715	7K20.1B	Monk total replacement of hip joint using cement
34997	7K20.1A	McKee total replacement of hip joint using cement
10341	7K21y00	Total prosthetic replacement hip joint not using cement OS
62092	7K20.1F	Turner total replacement of hip joint using cement
52901	7K20.16	Freeman total replacement of hip joint using cement
53109	7K21.11	Freeman total replacement of hip joint not using cement
56215	7K20.19	Ilch total replacement of hip joint using cement
66139	7K20.15	Farrer total replacement of hip joint using cement
73951	7K21.16	Ring total replacement of hip joint not using cement
51519	7K21.15	Monk total replacement of hip joint not using cement
94273	7K20.1D	Pretoria total replacement of hip joint using cement
96435	7K21.13	Lord total replacement of hip joint not using cement

Medical diagnosis codes for revision hip replacement

medcode	readcode	readterm
48220	7K22100	Conversion to total prosthetic replacement of hip joint NEC
31843	7K20100	Conversion to cemented total hip replacement
50624	7K21100	Conversion to uncemented total hip replacement
2032	7K22200	Revision of total prosthetic replacement of hip joint NEC
8895	7K20200	Revision cemented total hip replacement
29101	7K21200	Revision uncemented total hip replacement
38942	7K23200	Revision cemented hemiarthroplasty of hip
41370	7K22300	Attention to total hip replacement NEC
41184	7K24200	Revision uncemented hemiarthroplasty of hip

36700	7K20x11	Removal prev cemented total prosthetic replacement hip joint
28784	7K69100	Revision of resurfacing arthroplasty
55662	7K20500	Revision of hybrid total hip replacement NEC
38791	7K22x00	Conversion from prev total pros replace hip joint NEC
45930	7K68300	Conversion to excision arthroplasty
66363	7K22211	Revision of hybrid total hip replacement NEC
97176	7K22112	Conversion to hybrid total hip replacement NEC
99651	7K22x12	Conver from hybrid total prosth hip joint replace NEC
67306	7K20600	Conver from hybrid total prosth hip joint replace NEC
67778	7K20x00	Conversion from cemented total hip replacement
62133	7K20400	Conversion to hybrid total hip replacement NEC

Medical diagnosis codes for knee replacement

medcode	readcode	readterm
5362	7K30.1V	TKR -Total prosthetic replacement of knee joint using cement
3414	7K30.00	Total prosthetic replacement of knee joint using cement
673	7K32z00	Other total prosthetic replacement of knee joint NOS
3973	7K32.12	TKR - Other total prosthetic replacement of knee joint
8555	7K32.00	Other total prosthetic replacement of knee joint
28048	7K32000	Primary total knee replacement NEC
20746	7K30000	Primary cemented total knee replacement
17471	7K31.00	Total prosthetic replacement of knee joint not using cement
9877	7K31.12	TKR - Total prosthetic replacement knee joint without cement
11225	7K37.00	Cemented unicompartmental knee replacement
10372	7K30y00	Total prosthetic replacement of knee joint using cement OS
8006	7K30z00	Total prosthetic replacement of knee joint using cement NOS
9817	7K38.00	Uncemented unicompartmental knee replacement
36343	7K37000	Primary cemented unicompartmental knee replacement
49053	7K31000	Primary uncemented total knee replacement
54343	7K38000	Primary uncemented unicompartmental knee replacement
37979	7K32y00	Other total prosthetic replacement of knee joint OS
58612	7K31z00	Total prosthetic replacement knee joint not using cement NOS
55470	7K39.00	Hybrid unicompartmental knee replacement
37950	7K39000	Primary hybrid unicompartmental knee replacement
46475	7K30.16	Charnley total replacement of knee joint using cement
61687	7K30.13	Attenborough total replacement of knee joint using cement
50829	7K31y00	Total prosthetic replacement knee joint not using cement OS
93344	7K30.11	Anametric total replacement of knee joint using cement
44775	7K30.18	Denham total replacement of knee joint using cement

54860	7K30.19	Freeman total replacement of knee joint using cement
55991	7K30.1P	Sheehan total replacement of knee joint using cement
44926	7K30.1R	Stanmore total replacement of knee joint using cement
83544	7K32011	Primary hybrid total knee replacement NEC
47301	7K30.1N	Polycentric total replacement of knee joint using cement
49813	7K30.17	Deane total replacement of knee joint using cement
63086	7K30.1T	Uci total replacement of knee joint using cement
49716	7K30.1S	Swanson total replacement of knee joint using cement
92246	7K30.1H	Liverpool total replacement of knee joint using cement
63802	7K30.1A	Geomedic total replacement of knee joint using cement
66707	7K30.1E	Herbert total replacement of knee joint using cement
70507	7K30.1Q	Shiers total replacement of knee joint using cement
71456	7K30.15	Cavendish total replacement of knee joint using cement
99912	7K30.1I	Manchester total replacement of knee joint using cement

Medical diagnosis codes for revision knee replacement

medcode	readcode	readterm
28784	7K69100	Revision of resurfacing arthroplasty
73075	7K30x00	Conversion from cemented total knee replacement
93435	7K32112	Conversion to hybrid total knee replacement NEC
97341	7K31x00	Conversion from uncemented total knee replacement
97400	7K37x00	Conversion from cemented unicompartmental knee replacement
69999	7K30100	Conversion to cemented total knee replacement
41820	7K32x00	Conversion from total knee replacement NEC
42259	7K32211	Revision hybrid total knee replacement NEC
62757	7K32100	Conversion to total knee replacement NEC
11847	7K32200	Revision of total knee replacement NEC

38073	7K39200	Revision hybrid unicompartmental knee replacement
47223	7K32411	Attention to hybrid total knee replacement NEC
61288	7K38200	Revision uncemented unicompartmental knee replacement
58980	7K37200	Revision cemented unicompartmental knee replacement
38740	7K30x11	Removal previous cemented total prosthetic replacement knee
41545	7K31200	Revision uncemented total knee replacement
48815	7K32x11	Removal previous total prosthetic replacement knee joint NEC
54756	7K32400	Attention to total knee replacement NEC
10553	7K30200	Revision cemented total knee replacement

Medical diagnosis codes for hip pain

medcode	readcode	readterm
286	N094K12	Hip pain
1330	N094512	Hip joint pain
33407	N094K00	Arthralgia of hip

Medical diagnosis codes for knee pain

medcode	readcode	readterm
9517	1M10.00	Knee pain
554	N094611	Knee joint pain
6044	N094M00	Arthralgia of knee
6166	N094W00	Anterior knee pain
10389	1M12.00	Anterior knee pain

Medical diagnosis codes for hip arthritis

medcode	readcode	readterm
7334	N06z511	Hip arthritis NOS
17561	N010511	Hip pyogenic arthritis
66483	N01zH00	Infective arthritis NOS, of hip

Medical diagnosis codes for knee arthritis

medcode	readcode	readterm
2852	N06z611	Knee arthritis NOS
62037	N03xB00	Arthritis associated with other disease, knee
56895	N01zK00	Infective arthritis NOS, of knee

Medical diagnosis codes for degeneration of the knee

medcode	readcode	readterm
17176	N072100	Degenerative lesion of articular cartilage of knee

Supplementary information:

Implant survivorship curves for THR and TKR

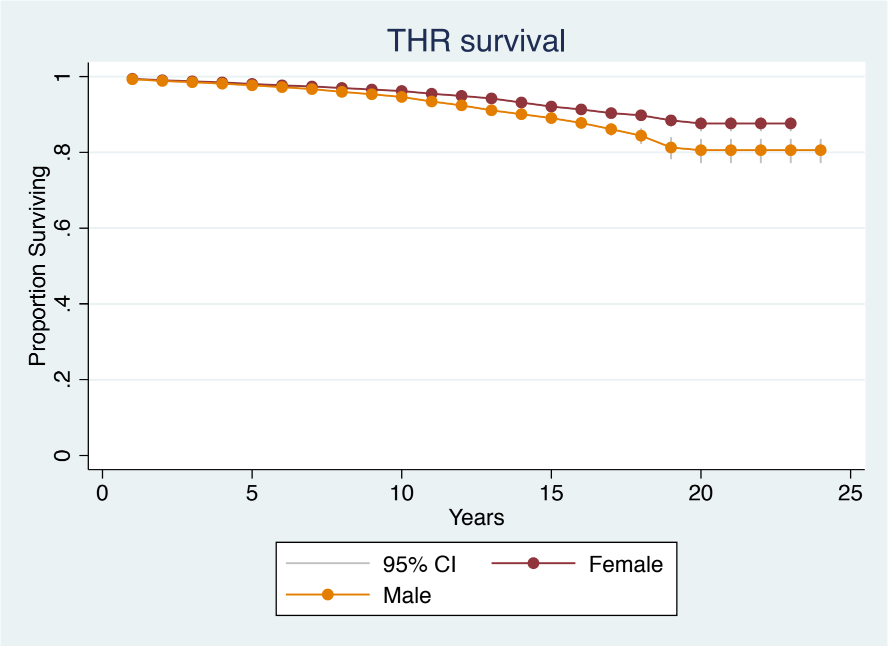

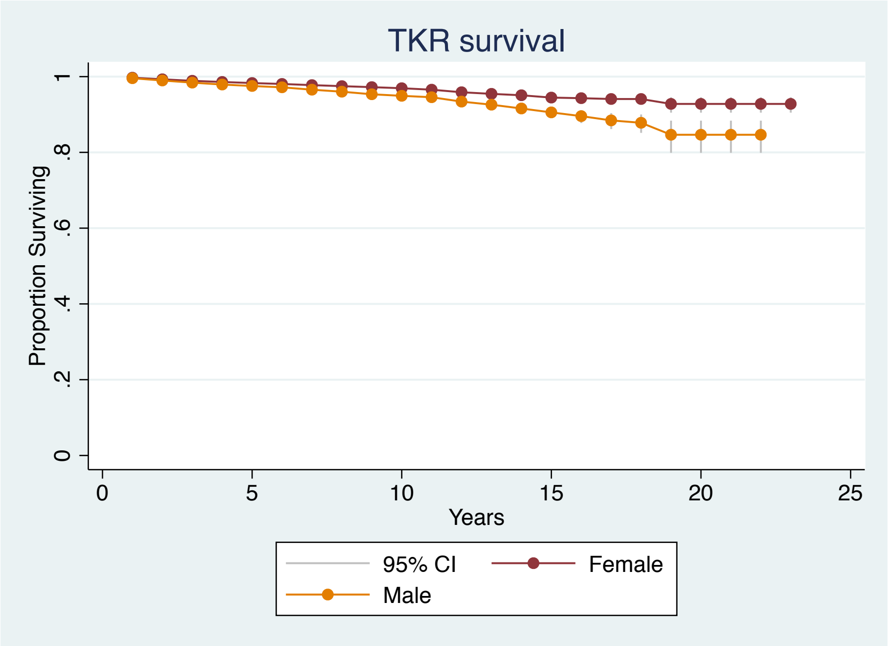

Supplement: Supplementary appendix [file mmc1.pdf]
